# Career Trajectories of Women From Underrepresented Minority Groups at an Academic Medical Center

**DOI:** 10.1001/jamanetworkopen.2021.2723

**Published:** 2021-03-25

**Authors:** Alyssa F. Westring, Mary D. Sammel, Rebecca M. Speck, Lucy Wolf Tuton, Jeane Ann Grisso, Stephanie B. Abbuhl

**Affiliations:** 1 Driehaus College of Business, DePaul University, Chicago, Illinois; 2 Colorado School of Public Health, University of Colorado Anschutz Medical Campus, Aurora; 3 Critical Path Institute, Tucson, Arizona; 4 Perelman School of Medicine, University of Pennsylvania, Philadelphia

## Abstract

This cohort study explores retention of women faculty members stratified by their race/ethnicity using data from a longitudinal study at 1 research-oriented institution.

## Introduction

Combined effects of the coronavirus disease 2019 pandemic and multiple high-profile events, such as the police killing of Breonna Taylor, have prompted a widespread reckoning with the systemic racial and gender inequalities that threaten the safety, livelihood, and well-being of women of color.^1^ In academic medicine, gender disparities in career advancement are well established; however, this work frequently sidelines conversations about racism and the double jeopardy facing women of color.^2^ Current Association of American Medical Colleges roster data show that, of full-time medical faculty, Black and Hispanic women together represent only 3.7% of faculty across ranks and 1.5% of full professors.^3^ Understanding career trajectories of women from groups underrepresented in medicine is important to developing strategies for the retention and promotion of a diverse academic faculty. Using data from a longitudinal study of women faculty at 1 research-oriented institution, we hypothesized that women from racial/ethnic minority groups were more likely to leave their assistant professor positions compared with White women.

## Methods

As part of a National Institutes of Health R01–funded cluster randomized intervention trial at 1 research-oriented institution, women assistant professors were recruited for participation in a study approved by the University of Pennsylvania institutional review board and tracked during 3 years.^4^ Participants provided written informed consent, their racial/ethnic identification, and personal and professional characteristics via a web-based survey in 2010. Three years later, as part of a secondary observational analysis on cohort attrition, participants reported their employment status at the institution. The primary randomized clinical trial followed the Consolidated Standards of Reporting Trials (CONSORT) reporting guideline.

In this cohort study, statistical analyses identified factors associated with attrition, including racial differences. A multivariable generalized linear model was used to estimate risk of attrition, using a generalized estimating equations framework to account for clustering by departments or divisions. The multivariable model included years in rank, marital status, and a validated measure of core self-evaluation.^5^ Analyses were conducted with Stata version 14 (StataCorp). Statistical significance was set at *P* < .05, and all tests were 2-tailed.

## Results

Of 178 eligible individuals, 134 women assistant professors (75.3%) from 27 departments and divisions participated in this study. Three years later, 131 faculty (98.5%) provided employment status at the institution. The racial composition was 15 individuals from groups underrepresented in medicine (11.5%; mean [SD] age, 40.5 [1.4] years), 37 Asian women (28.2%; mean [SD] age, 41.0 [0.9] years), and 79 White women (60.3%; mean [SD] age, 41.1 [0.6] years). Of the faculty from groups underrepresented in medicine, two-thirds (10 of 15 [66.7%]) were Black women. Overall, 21 faculty members (15.6%) departed from the institution during the 3 years: 6 of 15 women (40.0%) from groups underrepresented in medicine, compared with 9 of 79 White women (11.4%). Twenty of the 21 women faculty (95.2%) who departed completed postexit surveys describing their new positions. Of those women, 17 (85.0%) accepted positions primarily as assistant professors in academic medical health centers affiliated with medical schools. The other 3 faculty (15.0%) moved to nonacademic tracks at large health systems. No racial differences in subsequent employment were discernable, given the small sample size. Participant characteristics by race/ethnicity are reported in Table 1.

**Table 1.** Characteristics of 131 Participants by Race/Ethnicity

Characteristic	Women, No. (%)			*P* value^a^
	URM (n = 15)	White (n = 79)	Asian (n = 37)	
Attrition	6 (40.0)	9 (11.4)	6 (16.2)	.02
Unmarried	2 (13.3)	13 (16.5)	5 (13.5)	.90
≥1 Child at home	11 (73.3)	62 (78.5)	25 (67.6)	.45
Track				
Clinician educator	13 (86.7)	54 (68.4)	25 (67.6)	.55
Research	2 (13.3)	13 (16.5)	6 (16.2)	
Tenure	0	12 (15.2)	6 (16.2)	
Part time	1 (6.7)	4 (5.1)	1 (2.7)	.84
Time as assistant professor, y				
<3	5 (33.3)	36 (45.6)	18 (48.7)	.25
3-6	8 (53.3)	23 (29.1)	8 (21.6)	
>6	2 (13.3)	20 (25.3)	11 (29.7)	
Age, mean (SD), y	40.5 (1.37)	41.06 (0.58)	40.97 (0.88)	.91^b^
Work time per week, mean (SD), h	56.00 (2.45)	59.13 (1.10)	61.33 (1.61)	.28^b^
Core self-evaluations, mean (SD)^c^	3.43 (0.14)	3.45 (0.06)	3.29 (0.90)	.34^b^

Abbreviation: URM, underrepresented in medicine.

^a^ *P* values from Pearson χ^2^ or Fisher exact test as appropriate.

^b^ *P* values from 1-way analysis of variance.

^c^ Scale of 12 items rated on a 5-point scale from 1 (strongly disagree) to 5 (strongly agree).

Table 2 provides multivariable model results. Women underrepresented in medicine were 3.3 times more likely to leave the institution than White women during the study (risk ratio, 3.30; 95% CI, 1.38-7.91; *P* = .007). Attrition did not differ between Asian and White women (risk ratio, 1.67; 95% CI, 0.75-3.74; *P* = .21). Low core self-evaluation scores and being unmarried were also independently associated with attrition.

**Table 2.** Risk Factors Associated With Attrition^a^

Factor	Risk ratio (95% CI)	*P* value
Race/ethnicity		
White	1 [Reference]	NA
URM	3.30 (1.38-7.91)	.007
Asian	1.67 (0.75-3.74)	.21
Marital status		
Married	1 [Reference]	NA
Unmarried	3.03 (1.24-7.37)	.02
Core self-evaluations	0.43 (0.22-0.86)	.02
Time as assistant professor, y		
<3	1 [Reference]	NA
3-6	1.30 (0.51-3.28)	.58
>6	0.15 (0.02-1.28)	.08

Abbreviations: NA, not applicable; URM, underrepresented in medicine.

^a^ All variables from Table 1 were considered in multivariable modeling and were retained if statistically significant (ie, *P* < .05) or when demonstrated to confound the association between race and attrition. Confounding was assessed with a backward selection process and was defined as a 10% or greater absolute change in the regression coefficient for either race indicator when the confounder was removed from the model.

## Discussion

The future of academic medicine depends on the ability to harness the talent of diverse faculty.^6^ Our study found that women assistant professors from groups that are underrepresented in medicine were 3 times more likely to leave their positions compared with White women during only 3 years. Although these findings represent 1 institution during a defined period, they signal a need for ongoing and targeted intersectional research on attrition in medicine. A limitation of this study is that the sample size was too small to allow a detailed analysis of subsequent employment by race. Regardless of the nature of postdeparture employment, these differences suggest a mandate for every institution to closely monitor and analyze attrition data. It is clear that the host institution lost full-time faculty and diversity with these departures, along with the investments made in recruitment and early faculty support. Indeed, the success of academic institutions, as well as the future of medical research and practice, hinges on the ability to develop and implement evidenced-based initiatives that nurture and advance the careers of all faculty.
